# 半量维奈克拉联合减量HA方案诱导治疗急性髓系白血病的疗效分析

**DOI:** 10.3760/cma.j.cn121090-20250516-00232

**Published:** 2026-04

**Authors:** 嶂松 阎, 晓慧 索, 观臣 白, 斌 张, 劲松 贺, 捷思 李, 述宁 魏, 秋玲 李, 凯奇 刘, 营昌 秘

**Affiliations:** 1 中国医学科学院血液病医院（中国医学科学院血液学研究所），血液与健康全国重点实验室，国家血液系统疾病临床医学研究中心，细胞生态海河实验室，天津 300020 State Key Laboratory of Experimental Hematology, National Clinical Research Center for Blood Diseases, Haihe Laboratory of Cell Ecosystem. Institute of Hematology & Blood Diseases Hospital, Chinese Academy of Medical Sciences & Peking Union Medical College, Tianjin 300020, China; 2 天津医学健康研究院，天津 301600 Tianjin Medical Health Research Institute, Tianjin 301600, China; 3 邯郸市中心医院血液科，邯郸 056000 Department of Hematology, Handan Central Hospital, Handan 056000, China; 4 泰安市中心医院血液科，泰安 271000 Department of Hematology, the Affiliated Taian City Central Hospital of Qingdao University, Taian 271000, China

## Abstract

回顾性分析2020年6月至2023年4月期间接受HAV方案诱导治疗的33例初诊急性髓系白血病（AML）患者的临床数据。HAV方案具体为维奈克拉（Ven）第0天100 mg；第1～14天200 mg，口服；高三尖杉酯碱（HHT）2 mg/m^2^，第3～9天，静脉滴注；阿糖胞苷50 mg/m^2^，第3～9天，静脉滴注。33例患者中男性占36.4％（12/33），中位年龄为60（14～67）岁。诱导治疗1个周期后的总有效率为90.9％，完全缓解（CR）率为87.9％。CR患者中79.3％达到可测量残留病（MRD）阴性的CR。诱导治疗期间无患者死亡。中性粒细胞恢复（≥1.0×10^9^/L）和血小板恢复（≥30×10^9^/L）的中位时间分别为10（5～12）d和10（3～13）d。截至2024年10月30日，预估3年总生存率、无事件生存率和无病生存率分别为68.2％（95％ *CI*：59.7％～76.7％）、66.7％（95％ *CI*：58.5％～74.9％）和73.6％（95％ *CI*：65.6％～81.6％）。HAV是一种对初诊AML患者高效且安全的诱导治疗方案。

## Abstract (en)

We retrospectively analyzed clinical data on newly diagnosed acute myeloid leukemia（AML）patients who received HAV treatment between June 2020 and April 2023. The HAV regimen includes venetoclax at 100 mg on day 0 and 200 mg from day 1 to 14, administered orally; homoharringtonine at 2 mg/m² from day 3 to 9, given by intravenous infusion; and cytarabine at 50 mg/m² from day 3 to 9, also administered by intravenous infusion. Among 33 patients, 36.4％（12/33）were male, with a median age of 60 years（range, 14–67 years）. The overall response rate after one cycle of induction therapy was 90.9％, and the complete response（CR）rate was 87.9％. Among the patients, 79.3％ achieved CR with measurable residual disease negativity. No patient died during induction therapy. The median recovery times of neutrophils（≥1.0×10⁹/L）and platelets（≥30×10⁹/L）were 10（5–12）days and 10（3–13）days, respectively. Until October 30, 2024, the estimated 3-year overall survival rate, event-free survival rate, and disease-free survival rate were 68.2％（95％ *CI*: 59.7％–76.7％）, 66.7％（95％ *CI*: 58.5％–74.9％）, and 73.6％（95％ *CI*: 65.6％–81.6％）, respectively. HAV is a highly effective and safe induction therapy regimen for patients with newly diagnosed AML.

近年来大量研究证明BCL-2抑制剂维奈克拉（VEN）为基础的联合方案显著改善了急性髓系白血病（AML）的疗效。VEN为基础的低强度治疗方案（维奈克拉与去甲基化药物或低剂量阿糖胞苷联合）显著提高了不适合强化疗（unfit）AML患者的完全缓解（CR）率和总生存（OS）率[1]–[4]。对于适合强化疗的患者，VEN与强化疗联合也提高了CR率和可测量残留病（MRD）转阴率[5]–[8]。Chua等[6]采用200 mg/d的VEN与IA “2+5”方案联合诱导治疗AML取得了较好的疗效。高三尖杉酯碱（HHT）为紫杉烷类缬氨酸脱氢酶抑制剂，HHT与阿糖胞苷组成的HA方案为国内AML一线诱导治疗方案。HA方案还可与柔红霉素、阿克拉霉素等联合，组成三药方案（如HAD、HAA），进一步提高疗效[9]–[12]。前期研究证明，HHT可抑制抗凋亡蛋白MCL-1、BCL-XL的表达，与VEN联合具有协同作用[13]–[14]，因此VEN联合HA可能改善AML疗效，我们针对初诊AML患者设计了半量VEN（200 mg/d）与减量的HA联合方案（HAV），现对接受HAV方案诱导治疗的33例AML患者进行疗效分析。

## 病例与方法

一、病例

本研究为多中心、回顾性队列研究，纳入2020年6月至2023年4月期间于中国医学科学院血液病医院、邯郸市中心医院、泰安市中心医院接受HAV方案诱导治疗的33例初诊AML患者。所有患者均符合2016年WHO AML诊断标准，治疗前均签署知情同意书。

二、治疗方案

1. 诱导治疗：HAV方案具体为VEN 100 mg第0天；200 mg第1～14天，口服；HHT 2 mg/m^2^，第3～9天，静脉滴注；阿糖胞苷50 mg/m^2^，第3～9天，静脉滴注。高白细胞患者前期可采用羟基脲或阿糖胞苷降白细胞，以减少肿瘤溶解综合征的发生风险，WBC≤25×10^9^/L时开始口服VEN。治疗过程中联合应用强CYP3A抑制剂（如伏立康唑、泊沙康唑等）时，VEN维持100 mg/d的剂量。骨髓抑制期允许应用G-CSF。诱导治疗无效，即未达CR、CR伴血液学不完全恢复（CRi）、部分缓解（PR）、形态学无白血病状态（MLFS）的患者换用其他挽救治疗方案。

2. 缓解后治疗：达CR或CRi的患者根据欧洲白血病网（ELN）和中国AML诊断治疗指南建议接受异基因造血干细胞移植（allo-HSCT）[9]，未行allo-HSCT的患者继续巩固强化、维持治疗。巩固强化治疗包括2个疗程VEN（400 mg/d，7 d）联合中剂量阿糖胞苷［1 g·m^−2^·（12 h）^−1^，3 d］、1个疗程DA“2+5”方案（柔红霉素+阿糖胞苷）和2个疗程HA方案（5 d）。巩固强化治疗后持续CR的患者接受至少4个疗程的维持治疗（阿扎胞苷100 mg/d，5 d）。

三、疗效评价

评价总有效率（ORR）、OS、无事件生存（EFS）及无病生存（DFS）。ORR为CR率、CRi率、PR率、MLFS率之和。OS时间定义为诱导治疗开始至任何原因的死亡或末次随访日；EFS时间定义为诱导治疗开始至治疗失败、复发、死亡或末次随访日（以先发生者为准）；DFS时间定义为自达到CR至复发、死亡或末次随访日。常规采用多参数流式细胞术或特异融合基因定量方法进行MRD监测[15]。诱导治疗结束后4周左右复查骨髓、评价疗效，疗效判断标准参照国际工作组的标准[16]。

四、随访及不良事件评估

采用电话联系或查阅病历的方式进行随访，末次随访日期为2024年10月30日。治疗相关不良事件的判断采用美国国立癌症研究所不良事件的常用术语标准（NCI CTCAE5.0）[17]。

五、统计学处理

采用SPSS25.0进行统计学分析，数值采用百分比（分类变量）或中位值（连续变量）的方式表示。根据Kaplan-Meier计算OS、EFS、DFS。

## 结果

一、患者的一般资料

共33例初诊AML患者采用HAV方案治疗，中位年龄60（14～67）岁，男性占36.4％（12/33）。治疗前中位WBC 15.0（1.1～290.6）×10^9^/L，中位ECOG评分2（0～3）分。根据2022 ELN预后分组标准，低风险组9例（27.3％）、中风险组7例（21.2％）、高风险组17例（51.5％）。患者基线特征详见表1。

**表1:** 33例采用半量维奈克拉联合减量HA方案诱导治疗的急性髓系白血病患者基线临床资料

临床特征	数值
年龄［岁，*M*（范围）］	60（14～67）
性别［例（％）］	
男	12（36.4）
女	21（63.6）
ECOG评分［*M*（范围）］	2（0～3）
WBC［×10^9^/L，*M*（*IQR*）］	15.0（1.1～290.6）
PLT［×10^9^/L，*M*（*IQR*）］	49（13～204）
ELN 2022风险分组［例（％）］	
低风险	9（27.3）
中风险	7（21.2）
高风险	17（51.5）
基因突变［例（％）］	
NPM1	5（15.2）
CEBPA（B-zip）	5（15.2）
FLT3-ITD	7（21.2）
RUNX1	7（21.2）
DNMT3A	8（24.2）
TET2	5（15.2）
IDH2	5（15.2）
ASXL1	6（18.2）
BCOR	4（12.1）
PTPN11	2（6.1）
TP53	1（3.0）
融合基因［例（％）］	
STAT5b::RARα	1（3.0）
KAT6A::CREBBP	1（3.0）
MLL-AF9	2（6.1）
CBFB::MYH11	2（6.1）
RUNX1::RUNX1T1	1（3.0）
阴性	23（69.7）
未查	3（9.1）

**注** HA：高三尖杉酯碱、阿糖胞苷；ECOG：美国东部肿瘤协作组；ELN：欧洲白血病网

二、治疗反应和生存

1个疗程诱导治疗后ORR为90.9％（30/33），其中CR 29例（87.9％），PR 1例（3.0％）。3例（9.1％）NR。根据ELN2022预后分组分析，低风险组CR率88.9％（8/9）、中风险组为100％（7/7）、高风险组为82.4％（14/17）。CR患者中79.3％（23/29）流式细胞术MRD转阴性。诱导治疗未取得CR的4例患者（3例NR、1例PR）经挽救治疗3例达CR；共8例患者接受allo-HSCT。截至2024年10月30日，中位随访20（2～40）个月，预估3年OS率、EFS率、DFS率分别为68.2％（95％ *CI*：59.7％～76.7％）、66.7％（95％ *CI*：58.5％～74.9％）、73.6％（95％ *CI*：65.6％～81.6％），详见图1。

**图1:**
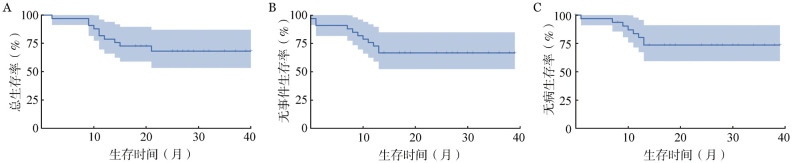
采用半量维奈克拉联合减量HA方案诱导治疗的急性髓系白血病患者总生存（A）、无事件生存（B）和无病生存（C）曲线

三、血液学不良反应

3级或更严重的血液学不良反应包括中性粒细胞减少（100％）、血小板减少（100％）。诱导治疗后WBC恢复至≥1.0×10^9^/L和PLT恢复至≥30×10^9^/L的中位时间分别为10（5～15）d和10（3～17）d。所有患者均未发生肿瘤溶解综合征。诱导治疗期间无死亡病例。

## 讨论

近年来，VEN联合去甲基化药物方案作为一线诱导治疗方案用于老年或不适合强化疗AML患者[1]–[3]以及与化疗药物联合用于适合强化疗AML患者[5]–[7]，均显著改善了患者临床疗效。但目前对VEN与化疗联合时，治疗方案的用药剂量、用药周期仍无一致的意见。HA方案是我国AML诱导治疗的一线推荐方案，HHT可抑制抗凋亡蛋白MCL-1、BCL-XL的表达，与VEN联合有可能发挥协同作用[13]–[14]。参考已报道的研究[6]，我们设计了半量VEN（200 mg/d，7 d）与减量的HA联合的方案，作为初诊AML患者的诱导治疗方案，以期提高临床疗效的同时减少治疗相关不良反应。

本研究共纳入33例患者接受该治疗方案。1个疗程诱导治疗后ORR为90.9％，CR率为87.9％。此外，通过流式细胞术检测发现，79.3％（23/29）患者达到CR且MRD转阴。预后良好组CR率88.9％（8/9）、预后中危组为100％（7/7）、高风险组为82.4％（14/17），这些结果与VEN联合强化疗的报道一致[5]–[8]。但需要强调的是，本方案中VEN的剂量（200 mg/d）虽然仅为现行常规剂量的一半，但其在高风险组中的疗效与研究报道的常规剂量相似[18]。另外，本研究与文献[6]结果均表明，VEN 200 mg/d剂量联合化疗均具有充足的安全性和有效性。这将有助于我们探索初诊AML诱导治疗中VEN的最佳剂量方案。

值得注意的是，本研究显示诱导治疗后WBC（≥1.0×10^9^/L）PLT（≥30×10^9^/L）的中位恢复时间分别为10（5～15）d和10（3～17）d，表明HAV治疗方案具有良好的安全性。与既往研究[5]–[8]相比，本研究的骨髓抑制期更短。骨髓抑制期的缩短可能与VEN剂量的降低有关，停止诱导治疗后4周左右评价疗效未观察到CRi患者，可能亦与此有关。

本研究数据显示，中位随访时间为20（2～40）个月，3年OS率、EFS率和DFS率分别达到68.2％（95％*CI*：59.7％～76.7％）、66.7％（95％ *CI*：58.5％～74.9％）和73.6％（95％ *CI*：65.6％～81.6％）。本组患者年龄偏高，中位年龄60岁，在仅有8例患者（24.2％）接受了allo-HSCT的情况下，这一结果令人鼓舞，值得深入探索。

本研究存在以下局限性：首先，为回顾性研究，入组患者数量较少（33例），缺乏随机对照组，样本存在选择偏倚，未能对伴不同基因突变、融合基因的患者进行亚组分析；其次，随访周期较短，为初步探索，不足以验证长期疗效。未来需要前瞻性、多中心临床研究进一步验证这一结果。

综上所述，半量VEN联合减量HA方案是初诊AML患者安全有效的诱导治疗方案。
